# Supplementary material for: Mapping the evolution of fertility support policies in China: A content and instrumental analysis
Source: PLoS One. 2025 Oct 9;20(10):e0332137. doi: 10.1371/journal.pone.0332137 (PMC12510515; doi:10.1371/journal.pone.0332137)
Supplement: S1 Appendix — (ZIP) [file pone.0332137.s001.zip › S1 Appendix. 226 original policy documents/6-国家人口和计划生育委员会、中国计划生育协会关于印发《计划生育村民自治规范》的通知(FBM-CLI-4-109244).doc]

国家人口和计划生育委员会、中国计划生育协会关于印发《计划生育村民自治规范》的通知

**发布部门：** [国家人口和计划生育委员会(含国家计划生育委员会)(已撤销)](https://www.pkulaw.com/law/chl?Aggs.IssueDepartment=60304) [中国计划生育协会](https://www.pkulaw.com/law/chl?Aggs.IssueDepartment=70572) [机构沿革](javascript:void(0))

**发文字号：**国人口发〔2008〕66号

**发布日期：**2008.09.25

**实施日期：**2008.09.25

**时效性：** [现行有效](https://www.pkulaw.com/law/chl?Aggs.TimelinessDic=01)

**效力级别：** [部门工作文件](https://www.pkulaw.com/law/chl?Aggs.EffectivenessDic=XE0303)

**法规类别：** [计划生育管理](https://www.pkulaw.com/law/chl?Aggs.Category=09705)

国家人口和计划生育委员会、中国计划生育协会关于印发《计划生育村民自治规范》的通知
（国人口发[2008]66号）

各省、自治区、直辖市人口计生委、计划生育协会，计划单列市、新疆生产建设兵团人口计生委、计划生育协会：
　　现将《计划生育村民自治规范》印发给你们，请遵照执行。
　　计划生育村民自治是稳定农村低生育水平、统筹解决人口问题的一项重要的基础性工作。各地要将其作为人口和计划生育工作新机制建设和社会主义新农村建设的一项重要内容，主动适应基层民主政治建设和行政管理体制改革的新形势，坚持依法办事、群众参与、因地制宜、循序渐进的原则，认真总结经验，把握阶段性特征和工作重点，逐步构建政府推动、民主管理、民主监督、维权自律的计划生育村民自治工作机制。
　　各级人口计生委、计划生育协会要积极争取党委、政府的支持，进一步创造条件，全面推进依法行政，建立健全村级人口和计划生育工作机构，落实专（兼）职计生干部报酬待遇和必要的工作经费；进一步加强对村级人口和计划生育工作的指导和帮助，定期组织培训，做好规范、评估，增强群众的自治能力；进一步转变政府职能，明确群众的自治权利，明确村民委员会的协助责任，实行"费随事转"，并自觉接受村民自治组织以及广大群众的监督。要按照本规范的要求，广泛动员、组织群众参与计划生育民主管理和民主监督，维护群众的合法权益，自觉实行计划生育。
　　各地可结合实际，认真组织开展计划生育村民自治合格村评估活动，切实防止发生政府推卸或转嫁责任的现象，切实防止发生重责任轻权利、重形式轻内容的现象，强化利益导向和优质服务，办实事求实效，逐步将计划生育村民自治引向深入。

人口计生委办公厅
二00八年九月二十五日

　　计划生育村民自治规范

　　为深入贯彻党的十七大关于坚持和完善基层群众自治制度以及《中共中央[国务院关于全面加强人口和计划生育工作统筹解决人口问题的决定](https://www.pkulaw.com/chl/5e7b01010a4102ffbdfb.html)》（中发［2006］22号）精神，推进基层计划生育民主管理和监督，保障群众知情权、参与权和监督权，维护群众的合法权益，依据《人口计生委民政部中国计划生育协会关于加强和完善村级计划生育民主管理和民主监督的意见》（国人口发〔2004〕85号），制定本规范。
　　**一、**民主管理
　　（一）建立健全村计划生育组织机构。村党支部、村民委员会负责领导村计划生育工作。配备计划生育专（兼）职干部，具体负责村计划生育日常工作。建立村计划生育协会网络，经常性地组织开展计划生育活动。计划生育专（兼）职干部经村计划生育协会理事会聘任，可兼任协会秘书长。各村民小组根据需要设立计划生育中心服务户长、育龄妇女小组长等。村计划生育协会按照章程建立会员小组，聘任协会会长、秘书长和会员小组长。
　　村计划生育组织机构由村民委员会根据有关法律法规、乡级以上政府或有关部门的要求，结合本村的实际，提出方案，由村民会议或村民代表会议表决通过。
　　（二）选举或选聘村计生专（兼）职干部。作为村民委员会成员的村专干，应按《[村民委员会组织法](https://www.pkulaw.com/chl/4613659a35cb0dc7bdfb.html)》规定，通过民主选举或民主协商的程序确定；不作为村民委员会成员的"县管乡聘村用"的村专（兼）职干部，乡（镇）选聘时应以适当的形式征求群众意见。各村民小组计划生育中心服务户长或育龄妇女小组长等，由村计划生育专（兼）职干部根据各村民小组多数育龄群众的意见提名，由村民委员会聘任。
　　不符合法律法规有关生育数量的规定生育的人员在一定时间内不得提名、聘任为村计划生育专（兼）职干部及村计划生育协会会长、秘书长，以及各村民小组计划生育中心服务户长、育龄妇女小组长和协会会员小组长等。
　　（三）制定章程公约。制定计划生育村民自治公约，或在村民自治章程中单设计划生育一章。章程公约的主要内容有：村民计划生育权利和义务，村计划生育协会的组织和活动，村民委员会以及村计划生育专（兼）职干部在计划生育工作中的职责和任务，民主管理和监督村级计划生育事务的主要事项及程序，村实施有利于计划生育的奖励优待措施，外出人员的计划生育服务和管理等。章程公约应符合法律法规及有关政策规定，简明具体，具有针对性和可操作性。
　　制定或修改章程公约的基本程序是：宣传发动群众，提高村民的权利意识和参与意识；在乡级政府的指导下，组织有关人员起草；征求村计划生育协会以及广大村民的意见和建议；报乡级政府进行合法性审查；召开村民会议或村民代表会议，表决通过；报乡级政府备案。
　　（四）签订计划生育合同。实行计划生育合同管理的地区应分类指导，按需组织实施合同管理。
　　首先，由村计划生育专（兼）职干部提出本村需签订计划生育合同的对象户名单。然后，有针对性地草拟合同或协议书文本。合同或协议书文本必须符合有关法律法规及政策规定，明确双方的权利和义务，但不得违法增加群众的义务；明确双方的违约责任，但不得设定违法收费和罚款的条款；明确合同争议的解决途径。最后，由对象户与村民委员会自愿签订。
　　（五）民主决策计划生育事务。利用村集体经济收入，建设计划生育基础设施、制定计划生育奖励优待措施、开展规模较大的计划生育/生殖健康活动、落实计划生育专（兼）职干部报酬待遇等涉及村民切身利益的重大事项，必须经村民会议或村民代表会议讨论决定。重要的计划生育服务和管理事项可"一事一议"。
　　计划生育重大事项原则上要遵循以下决策程序：由村党组织、村民委员会、计划生育协会、村集体经济组织、1/10以上村民联名或1/5以上村民代表联名提出议案；召开村党组织和村民委员会联席会议，研究提出具体意见或建议；对需要提交村民会议或村民代表会议讨论决定的计划生育事项，应及时向村民公告，广泛征求意见；召开村民会议或村民代表会议讨论决定；会后应及时公布表决结果。村民会议或村民代表会议讨论决定的事项，要形成书面记录并妥善保存。计划生育"一事一议"由村计划生育专（兼）职干部或委托村计划生育协会，在广泛征求群众意见的基础上提交村民委员会讨论决定。
　　除发生自然灾害等紧急情况外，村民会议或村民代表会议依法形成的有关计划生育的决议不得随意更改，如因情况发生变化、确需更改的，要通过村民会议或村民代表会议讨论决定。
　　（六）评议计划生育奖励与扶助对象（户）。各级政府或有关部门组织实施的农村部分计划生育家庭奖励扶助制度、"少生快富"工程、特别扶助制度以及各类计划生育帮扶、救助工作的对象、项目户等，应根据政策或项目的具体要求，进行群众评议。
　　具体程序是：公开、深入地宣传有关政策或项目，让广大群众充分知晓；群众根据要求和本人（户）的具体情况，提出申请；计划生育专（兼）职干部或委托村计划生育协会按要求组织部分有代表性的群众进行资格评议，对群众提交的申请进行初步审核并提出意见；村民委员会根据群众评议意见提出审议意见；将审议结果及时向全体村民公示；公示期满，及时将符合项目或政策要求的对象（户）以及村民委员会的意见，报送乡级政府有关办事机构。对提出申请但不符合要求的，要及时反馈情况并说明原因。
　　（七）组织开展人口和计划生育宣传教育及服务活动。因地制宜设立村计划生育服务室、人口学校及协会会员之家。协助乡级政府及有关部门开展人口和计划生育法律法规和政策、优生优育和生殖健康知识以及新农村文明风尚的宣传，协助组织开展计划生育、优生优育以及生殖健康服务；根据本村的经济情况、多数育龄群众的需要以及计划生育困难家庭的情况，自主组织开展有关宣传教育及服务活动，并对计划生育困难家庭给予重点帮扶。
　　应主动了解本村育龄群众的计划生育/生殖健康的需要，依靠村计划生育协会，以群众喜闻乐见的活动为载体，自主组织开展有关宣传教育和服务活动。多方筹集经费，开展帮扶活动。以村民小组、育龄妇女小组或计划生育协会会员小组等为单位，民主讨论决定重点帮扶的计划生育困难家庭。制定适当激励措施，增强村民小组、育龄妇女小组、协会会员小组等的凝聚力，提高群众参与的积极性。

　　**二、**民主监督
　　（八）公开计划生育事务。凡是群众普遍关心、涉及群众切身利益、需要群众监督的计划生育事项都要公开。人口和计划生育相关的政策法规，公民实行计划生育应享有的权利和义务，人口和计划生育办事规则与程序，以及工作纪律和维权举报电话等内容要长期公开；本村再生育申报、生育、违法生育处理、奖励和扶助制度落实等情况要定期公开。在固定的村务公开栏中要有一定的版面，用于公开计划生育事项，同时还可以通过广播、电视、宣传品等其他有效形式公开。村务公开要注意保护个人隐私。
　　由村计划生育专（兼）职干部根据多数群众的意见和建议，提出拟公开的具体内容和形式；村务公开监督小组审查同意；村民委员会确定公开的事项、时间及方式；最后，由村计划生育专（兼）职干部具体负责实施计划生育村务公开。
　　（九）应答群众计划生育投诉和质询。村设立意见箱或计划生育意见箱。村民对村级计划生育事务以及其他人口和计划生育工作，特别是村务公开的内容有异议的，可以口头或书面形式向村党组织、村民委员会、计划生育专（兼）职干部、村计划生育协会、村务公开监督小组以及其他有关组织投诉和质询。
　　村计划生育专（兼）职干部应及时做好群众投诉、质询登记，做好调查研究，并向村民委员会提出建议。对群众的投诉或质询，村民委员会能解决的应及时予以解决，并由村计划生育专（兼）职干部向群众反馈；不能及时解决的，应在10日内向群众作出解释，并对将来可以解决的问题，提出解决的时限。对涉及各级政府及有关部门的问题，应及时向乡级政府及有关部门反映。计划生育专（兼）职干部每季度统计分析一次本村群众的投诉、质询情况，并向村民委员会报告。
　　（十）民主评议村计划生育工作。评议的主要内容是村民委员会和村专（兼）职干部计划生育履职、人口和计划生育法律法规和乡级以上政府或部门的有关政策措施落实、计划生育事务公开、人口计生工作中有无弄虚作假、村干部带头实行计划生育、听取群众意见及工作改进、群众参与和满意程度等方面的情况。
　　评议工作在乡级政府领导下组织实施。主要以村民委员会向村民会议或村民代表会议报告工作、走访座谈、问卷调查等形式进行。民主评议可根据需要每年组织1－2次，其结果作为乡级政府对村计划生育工作评价的主要依据。
　　（十一）发挥计划生育协会的民主监督作用。支持协会广泛参与村计划生育服务和管理，代表村民监督村民委员会以及各级政府和有关部门的人口和计划生育工作，反映群众呼声，提出工作意见和建议。计划生育协会应通过理事访问、会员小组活动等多种渠道了解，并及时反映群众的需求和意见。
　　（十二）依法追究决策责任。未经村民会议或村民代表会议讨论决定，任何组织或个人利用集体经济收入实施的有关计划生育的重大项目和活动，村民有权拒绝参与，造成的损失由责任人承担。对不称职的村计划生育专（兼）职干部，要按照法律法规及有关规定，依法进行撤换。
　　附件：

计划生育村民自治合格村评估内容和指标

　　**一、**村计划生育工作组织机构健全。计划生育专（兼）职干部、村民小组中心户长或育龄妇女小组长等职责、任务明确。村计划生育协会网络健全。

　　**二、**计划生育村民公约或自治章程符合国家有关法律、法规和政策，并体现大多数村民的意志。实行合同管理的地方，村民知情自愿与村民委员会签订计划生育合同或协议，其内容合法、合理、互约，合同双方的权利义务对等。

　　**三、**能较好地落实村民公约或自治章程规定的计划生育奖励优惠政策和措施。村集体资源的分配、使用符合人口和计划生育政策法规的有关规定，能较好保护实行计划生育的个人和家庭利益。

　　**四、**经常组织开展人口和计划生育宣传教育及服务活动。育龄群众对人口和计划生育政策法规和生殖健康知识的知晓率达到80％以上。个人隐私权得到保护。

　　**五、**组织开展对计划生育困难家庭的重点帮扶、救助活动，并有较明显效果。

　　**六、**全面、真实、及时公开人口和计划生育政策法规及落实情况等重大事项。

　　**七、**多数育龄群众参与民主评议本村计划生育工作情况。评议结果与村民委员会及村计划生育专（兼）职干部报酬待遇挂钩。

　　**八、**能及时妥当处理村民通过各种渠道反映的问题。没有违法的收费和罚款。近三年无因计划生育问题引起的村民集体上访和严重违法事件。

　　**九、**近三年已婚育龄妇女综合避孕率达到80%，符合政策生育率达到85%，没有非医学需要胎儿性别鉴定和选择性别人工终止妊娠行为，无溺、弃女婴事件，无大月份引产。

© 北大法宝：（[www.pkulaw.com](https://www.pkulaw.com/)) 专业提供法律信息、法学知识和法律软件领域各类解决方案。北大法宝为您提供丰富的参考资料，正式引用法规条文时请与标准文本核对。
欢迎查看所有[产品和服务](https://www.pkulaw.com/helps/2.html)。法宝快讯：[如何快速找到您需要的检索结果？](https://www.pkulaw.com/helps/70.html)[法宝](https://www.pkulaw.com/helps/69.html)V6有何新特色？

扫描二维码阅读原文

**原文链接：**<https://www.pkulaw.com/chl/e08729a8936f6d2fbdfb.html>
